# Highly Sensitive and Selective Colorimetric Detection of Methylmercury Based on DNA Functionalized Gold Nanoparticles

**DOI:** 10.3390/s18082679

**Published:** 2018-08-15

**Authors:** Zheng-Jun Xie, Xian-Yu Bao, Chi-Fang Peng

**Affiliations:** 1 State Key Laboratory of Dairy Biotechnology, Shanghai Engineering Research Center of Dairy Biotechnology, Dairy Research Institute, Bright Dairy & Food Co., Ltd., Shanghai 200436, China; xiezj@jiangnan.edu.cn; 2 School of Food Science and Technology, Jiangnan University, Wuxi 214122, China; 6150111037@vip.jiangnan.edu.cn; 3 Shenzhen Academy of Inspection and Quarantine, Shenzhen 518045, China

**Keywords:** methylmercury, gold nanoparticles, enzyme mimic, chromogenic reaction

## Abstract

A new colorimetric detection of methylmercury (CH_3_Hg^+^) was developed, which was based on the surface deposition of Hg enhancing the catalytic activity of gold nanoparticles (AuNPs). The AuNPs were functionalized with a specific DNA strand (H_T7_) recognizing CH_3_Hg^+^, which was used to capture and separate CH_3_Hg^+^ by centrifugation. It was found that the CH_3_Hg^+^ reduction resulted in the deposition of Hg onto the surface of AuNPs. As a result, the catalytic activity of the AuNPs toward the chromogenic reaction of 3,3,5,5-tetramethylbenzidine (TMB)-H_2_O_2_ was remarkably enhanced. Under optimal conditions, a limit of detection of 5.0 nM was obtained for CH_3_Hg^+^ with a linear range of 10–200 nM. We demonstrated that the colorimetric method was fairly simple with a low cost and can be conveniently applied to CH_3_Hg^+^ detection in environmental samples.

## 1. Introduction

Mercuric ions widely exist in the environment and have distinct toxic effects on human beings. Organic forms of mercury (Hg) demonstrate much higher toxicity than inorganic Hg due to their higher lipophilicity and easier bioaccumulation through the food chain, such as in the tissue of fish [1,2]. The main organic species of mercury, methylmercury (CH_3_Hg^+^), has been recognized as a potent neurotoxin that causes damage to the brain and nervous system [1,3]. Due to the severe effects of mercury, the U.S. Environmental Protection Agency has set a maximum level (10 nM, 2 ppb) for mercury species in drinking water [1]. Usually, complex hyphenated techniques, such as high performance liquid chromatography (HPLC) or gas chromatography (GC), coupled to specific detectors, such as mass spectrometry (MS), inductively coupled plasma mass spectrometry (ICP-MS) or atomic fluorescence spectrometry (AFS), are required for methylmercury detection [4,5,6]. However, these techniques generally require expensive instruments and are time-consuming and costly. To overcome the limitation of the above methods, recently, nanomaterial-based assays have been widely used for developing rapid and cost-effective methods for the detection of various heavy metal ions in environmental and biological samples [7,8,9]. Due to the strong metallophilic interactions between Hg^2+^ and some other metallic atoms, such as gold and silver, numerous metallic nanoparticle-based assays for Hg^2+^ have been developed [10,11,12,13,14,15,16]. However, there are fewer nanomaterial-based assays for CH_3_Hg^+^ compared to Hg^2+^ ions [17,18,19], which is probably due to the weak interactions between CH_3_Hg^+^ and metal nanomaterials.

Only a few studies have reported the development of nanomaterial-based detection methods for CH_3_Hg^+^. For example, Chen at el. developed a colorimetric nanosensor for mercury speciation, which was based on the analyte-induced aggregation of gold nanoparticles (Au NPs) with the assistance of a thiol-containing ligand of diethyldithiocarbamate (DDTC) [18]. Pandeeswar et al. presented a novel optoelectronic approach for detection of Hg^2+^ and CH_3_Hg^+^, which was based on nanoarchitectonics that consists of an adenine (A)-conjugated small organic semiconductor (BNA) and deoxyribo-oligothymidine (dTn) [20]. However, this device cannot distinguish Hg^2+^ from CH_3_Hg^+^. Recently, Deng et al. reported that a DNA strand, H_T7_, can bind to CH_3_Hg^+^ with a higher *K*_b_ value of (5.57 ± 0.47) × 10^6^ M^−1^ compared to that of Hg^2+^ ((1.51 ± 0.18) × 10^6^ M^−1^) [19]. Based on this, they were able to discriminate between CH_3_Hg^+^ and Hg^2+^ ions by forming Ag/Hg amalgam with a CH_3_Hg^+^-specific fluorophore-labeled DNA probe and fabricated a highly selective fluorescent assay for CH_3_Hg^+^. More recently, Yang et al. designed a specific visual detection method for CH_3_Hg^+^ and ethylmercury based on DNA-templated alloy Ag/Au NPs [21]. However, this visual detection method for methylmercury and ethylmercury requires subtle temperature adjustments and its sensitivity was above the micromolar level. Thus, developing a simple and selective colorimetric assay for CH_3_Hg^+^ is still an important and difficult task.

Recently, some methods for the detection of Hg^2+^ were reported, which were based on the peroxidase-like activity of the AuHg alloy NPs. For example, Long et al. [22] found that AuNPs possess excellent peroxidase-like activity after the deposition of Hg^2+^ onto the surface of AuNPs. The peroxidase-like activity enhancement of AuNPs, after Hg^0^ deposition onto the surface of AuNPs, was suggested to be the result of the accelerated decomposition of H_2_O_2_ and the stabilization of hydroxyl radicals on the surface of AuNPs. This phenomenon can be applied in the development of colorimetric and fluorescent assays for Hg^2+^ [22,23,24]. Our group also reported that catalytic DNA-AuNPs and DNA-Ag/Pt nanoclusters can be used to detect Hg^2+^ with high selectivity and sensitivity by stimulating or inhibiting their peroxidase-like activity [25,26]. Interestingly, compared with citrate stabilized AuNPs, AuNPs functionalized with a T-rich DNA strand can obviously improve the selectivity and can simplify the sample pretreatment for the colorimetric detection of Hg^2+^ [25]. However, to the best of our knowledge, there is no report on the application of nanomaterial enzyme mimics in CH_3_Hg^+^ detection. Herein, we found that CH_3_Hg^+^ captured by the AuNPs functionalized with CH_3_Hg^+^-specific DNA strands can be reduced by NaBH_4_. This results in Hg deposition onto the surface of AuNPs, thus stimulating the peroxidase-like activity of the AuNPs. Based on this finding, a highly sensitive and selective colorimetric assay for CH_3_Hg^+^ was developed.

## 2. Materials and Methods

### 2.1. Chemicals and Materials

HAuCl_4_, CH_3_Hg^+^Cl, NaBH_4_, 3,3,5,5-Tetramethylbenzidine (TMB) and H_2_O_2_ (30%) were purchased from Aladdin Reagent (Shanghai, China). The single-strand oligonucleotides were obtained from Sangon Biotech (Shanghai, China) and the sequences of these DNA strands were listed in Table 1. Hg(NO_3_)_2_ and all the other metal salts were purchased from the National Institute of Metrology (Beijing, China). All of the reagents used were of analytical grade. Ultra-pure water prepared with a Milli-Q Pure system was used for all experiments.

### 2.2. Synthesis of AuNPs and the Modification by DNA Strands

The AuNPs were prepared through the citrate-mediated reduction of HAuCl_4_ [24]. Briefly, HAuCl_4_ (0.01%, 100 mL) was added to a flask, which had been washed with aqua regia and ultra-pure water. After the solution was heated to boiling, sodium citrate (1.0%, 2.0 mL) was quickly added with stirring. When we observed a color change in the mixture to wine red, the mixture was further boiled for another 5 min and cooled to room temperature. The diameter of AuNPs was about 15 nM and their concentration was estimated to be 3 nM.

The DNA modification of the AuNPs was achieved by directly incubating thiolated single-strand DNA (H_T7_) with the AuNPs. Briefly, the AuNPs (3 nM, 990 μL) and thiolated DNA (100 μM, 5 μL) were mixed together and incubated at an ambient temperature for 24 h. After this, the mixture was centrifuged for 15 min at 10,000× *g* rpm and excessive DNA strands were removed. After repeating the centrifugation once, the obtained DNA-AuNPs complex was resuspended in phosphate buffer (10 mM, pH of 7.0) and stored at 4 °C.

### 2.3. Colorimetric Detection of CH_3_Hg^+^

To 25 μL of DNA-AuNPs complex (0.6 nM), 175 μL of Tris-HNO_3_ buffer (5.0 mM, pH 7.0) and 500 μL of CH_3_Hg^+^ solution at different concentrations were added. After being incubated for 10 min, the mixtures were centrifuged at 10,000 rpm for 15 min and the supernatants were discarded. To the 50 μL of retained mixture, we added 50 μL of NaBH_4_ (1.0 mM). After being incubated for another 10 min, 90 μL of citrate buffer (100 mM, pH 4.5), 100 μL of TMB (1.5 mM) and 60 μL of H_2_O_2_ (1.5 M) were transferred into the solution. The catalytic reaction was subsequently recorded at 650 nm by a microplate reader (PowerWave XS_2_, Bio-Tek, Winooski, VT, USA) after 10 min. For detection of CH_3_Hg^+^ in lake water, the samples were filtered through microfiltration membranes and measured by the above method.

## 3. Results and Discussion

### 3.1. Characterization of AuNPs and DNA-AuNPs Complex

**Figure 1:**
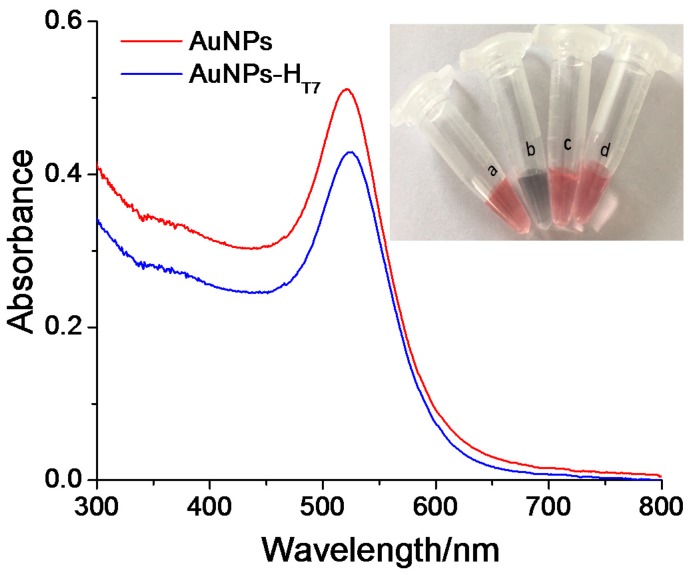
UV–vis spectra of AuNPs and AuNPs-H_T7_. The inset shows the photographs of (a) AuNPs, (b) AuNPs with 0.15 mol/L NaCl, (c) AuNPs-H_T7_ and (d) AuNPs-H_T7_ with 0.15 mol/L NaCl.

Figure 1 shows that the UV–vis absorption spectra of the AuNPs has a maximum absorption peak (λ_max_) at 520 nm. After the AuNPs were modified with H_T7_, which is a CH_3_Hg^+^ recognition DNA strand, the λ_max_ of the AuNPs experienced a red shift to 522 nm. This result suggested that the DNA-AuNPs complex (H_T7_-AuNPs) was obtained. The H_T7_-AuNPs complex was stable in 0.15 M NaCl (the inset in Figure 1), which also indicated the successful preparation of the H_T7_-AuNPs.

### 3.2. Colorimetric Detection of CH_3_Hg^+^

**Figure 2:**
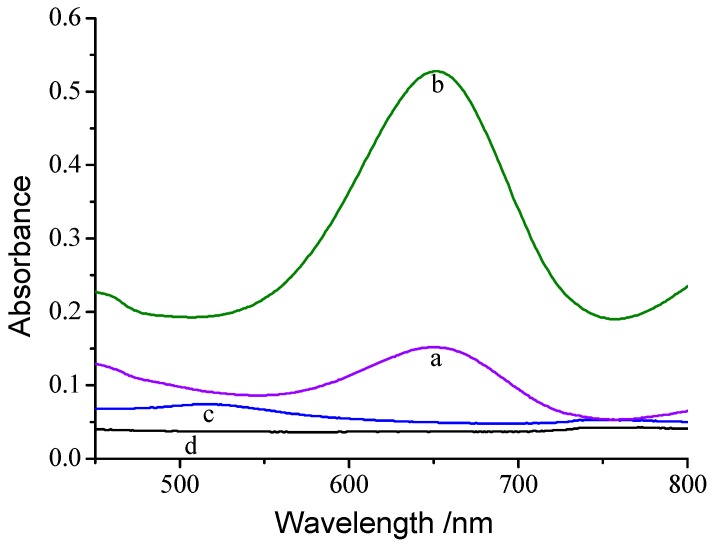
UV–vis spectra of AuNPs-H_T7_ + TMB-H_2_O_2_ reaction solution (a) before and (b) after capturing CH_3_Hg^+^ and Hg deposition, (c) AuNPs-H_T7_ solution and (d) TMB-H_2_O_2_ substrate.

**Scheme 1:**
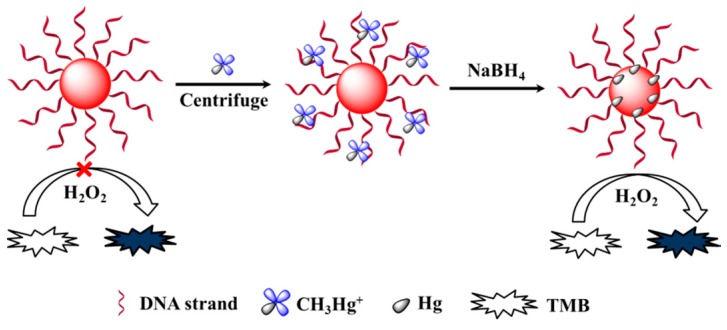
CH_3_Hg^+^ sensing mechanism.

As shown in Figure 2, AuNPs-H_T7_ demonstrated weak catalytic activity and we only found a weak signal with a peak at 650 nm. After being captured by the H_T7_ strand, CH_3_Hg^+^ species can be deposited onto the surface of AuNPs through Au/Hg amalgamation since they can be reduced to Hg^0^ by NaBH_4_ [19,27,28]. In the above process, the catalytic activity of AuNPs-HT_7_ was obviously increased, which was supported by the appearance of a strong signal at 650 nm. This was due to the oxidation of TMB by the hydroxyl radical that is stabilized on the surface of AuNPs, which produces a blue one-electron oxidation product (i.e., cation free-radical, TMB^+^) [29]. The reaction is shown in Figure S1. This change in peroxidase-like activity of the AuNPs suggests the deposition of Hg onto the surface of AuNPs [22,25]. The CH_3_Hg^+^ sensing mechanism is depicted in Scheme 1.

**Figure 3:**
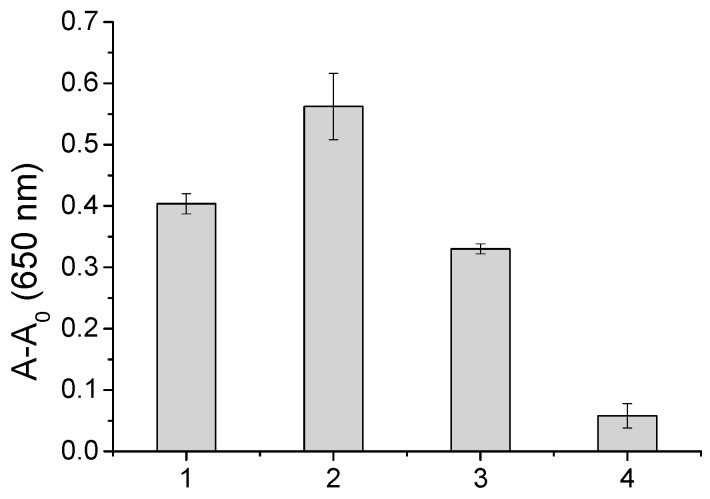
Effect of DNA sequence on the colorimetric detection for CH_3_Hg^+^. AuNPs, 0.6 nM; CH_3_Hg^+^, 100 nM. TMB, 1.0 × 10^−3^ M; H_2_O_2_, 1.5 M; pH, 4.5; and incubation time, 25 min.

Since the number of T-T pairs may affect the response of the AuNPs-ssDNA complex to CH_3_Hg^+^, H_T5_, H_T7_ and H_T9_ strands were used to modify the AuNPs, respectively, before we carried out a comparison of these AuNPs-ssDNA complexes. These AuNPs-ssDNA complexes were also characterized by UV–vis spectra, which demonstrated the same change compared with the AuNPs. As shown in Figure 3, H_T7_ modified AuNPs demonstrated more sensitive responses to CH_3_Hg^+^ compared to H_T5_, H_T9_ or H_R_ modified AuNPs. This result suggested that the higher affinity of DNA strand to CH_3_Hg^+^ over Hg^2+^ was still the main factor determining the selectivity of this probe [19].

**Figure 4:**
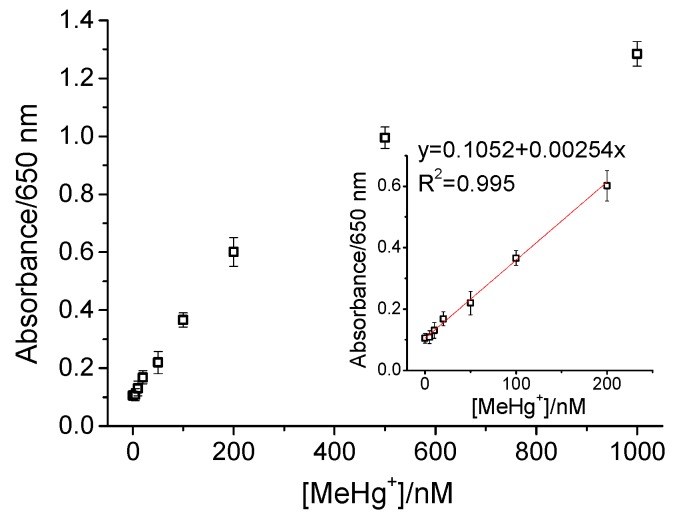
Calibration curve for the detection of CH_3_Hg^+^. AuNPs, 6.0 × 10^−10^ M; TMB, 1.5 × 10^−3^ M; H_2_O_2_, 1.5 M; Hg^2+^, 4.0 × 10^−7^ M; pH, 4.4; and incubation time, 20 min.

The enhanced peroxidase-like activity of AuNPs caused by CH_3_Hg^+^ was further applied in the development of a colorimetric assay for CH_3_Hg^+^. As shown in Figure 4, the absorbance increased as the CH_3_Hg^+^ concentration increased in the range of 0–1000 nM. A good linear relationship between CH_3_Hg^+^ concentration and absorbance values can be obtained in the range of 10–200 nM. The limit of detection (3-fold signal to noise, S/N = 3) was evaluated to be 5.0 nM.

**Figure 5:**
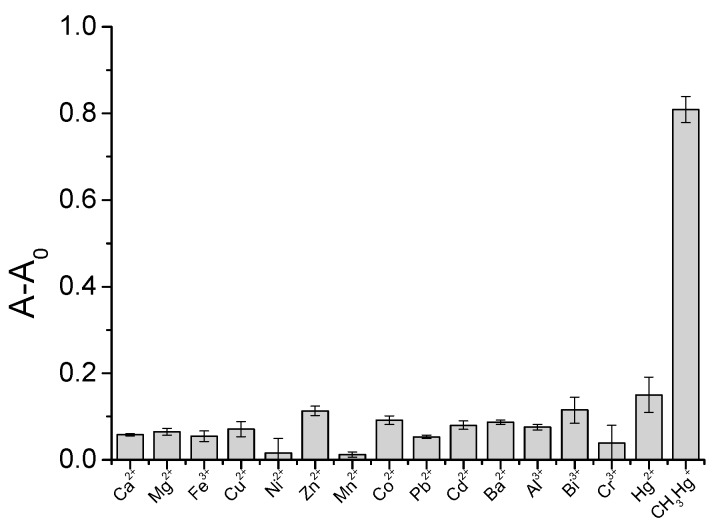
Selectivity of the colorimetric assay for CH_3_Hg^+^.

Some common metal ions were tested in this colorimetric assay. As shown in Figure 5, most of common metal ions at a 20-fold higher concentration and the same concentration of Hg^2+^ showed very weak responses. On the contrary, when citrate-stabilized AuNPs were incubated with Hg^2+^ or CH_3_Hg^+^ ions, almost the same catalytic enhancement of AuNPs was observed (Figure S2). The above results clearly demonstrated the good selectivity of this colorimetric assay for CH_3_Hg^+^, which was mainly due to the two aspects: (1) CH_3_Hg^+^-specific DNA scaffold has much higher affinity to CH_3_Hg^+^ (a *K*_b_ value of (5.57 ± 0.47) × 10^6^ M^−1^) compared to Hg^2+^ ((1.51 ± 0.18) × 10^6^ M^−1^); and (2) the centrifugation and separation of AuNPs-H_T7_ enriched CH_3_Hg^+^ over Hg^2+^. It also should be pointed out that Hg^2+^ has good affinity with AuNPs. However, the DNA strand on the AuNPs will interact with Hg^2+^ and thus, will eventually affect the deposition of Hg^0^. In this case, the H_T7_ strands probably hindered the deposition of Hg^2+^.

The sensitivity of the proposed method was higher than the two typical colorimetric methods [18,21] and comparable with some typical nanosensors or chemosensors (Table 2) [1,17,30,31]. However, the selectivity of this method needs to be further improved when compared with the established colorimetric [21] and fluorescent methods [19,30].

The real water samples from the Li Lake in Wuxi, Jiangsu Province were obtained and spiked with different concentrations of CH_3_Hg^+^ (20 nM, 50 nM and 100 nM). As shown in Table 3, the recovery of the added CH_3_Hg^+^ with the colorimetric method was in the range of 93.6–102.1%, which demonstrated the reliability of this assay for the detection of CH_3_Hg^+^ in real samples.

## 4. Conclusions

In summary, we developed a highly sensitive and selective colorimetric method for the detection of CH_3_Hg^+^, which was based on the surface deposition of Hg enhancing the catalytic activity of AuNPs. The limit of detection was 5.0 nM with a linear range of 10–200 nM. This colorimetric method has potential in the detection of CH_3_Hg^+^ in environmental samples since it also demonstrated other advantages of being simple, rapid and cost-effective. However, this method needs to be further improved with respect to its selectivity to Hg^2+^. This probably can be further improved through adopting magnetic core gold shell nanocomposites due to their more convenient separation and enrichment capability.

## Figures and Tables

**Table 1:** Oligonucleotide Sequences Used in This Work ^a^.

Type	Sequence
H_T5_	5′-SH-CTTTGTTAAAAATTCTTTG-3′
H_T7_	5′-SH-GTTCTTTGTTAAAAATTCTTTGTTC-3′
H_T9_	5′-SH-TTGTTCTTTGTTAAAAATTCTTTGTTCTT-3′
H_R_	5′-SH-CTGCTGCTGCAAAAAGCAGCAGCAG-3′

^a^ H_T5_, H_T7_ and H_T9_ represent CH_3_Hg^+^-specific DNA with different T bases, while H_R_ represents random DNA.

**Table 2:** Summarize of some typical method of CH_3_Hg^+^.

Method	Probe	Limit of Detection	Linear Range	Selectivity to Hg^2+^	Sample	Ref.
Fluorescent	Lys VI-AuNCs	CH_3_Hg^+^: 3 pM Hg^2+^: 4 nM	CH_3_Hg^+^: 15–500 nM; Hg^2+^: 10−5000 pM		seawater	[1]
Upconversion fluorescence	hCy7-UCNPs	0.8 ppb	0–7 μM;	Not clear	cells	[17]
Colorimetric	Diethyldithiocarbamate-AuNPs	CH_3_Hg^+^: 15 nM Hg^2+^: 10 nM	CH_3_Hg^+^: 0.03–0.8 μM; Hg^2+^: 0.01–0.1 μM	EDTA can mask Hg^2+^	drinking water	[18]
Fluorescent sensing by in-situ synthesis	carbon dots	5.9 nM	23–278 nM	tolerate with 250-fold Hg^2+^	River/sea water ^a^	[30]
Fluorescent sensing by in-situ synthesis	Silver nanocluster	0.4 nM	2.0 nM–12.0 μM	tolerate with 50-fold Hg^2+^	Fish sample	[19]
chiro-optical	adenine -small organic semiconductor and oligothymidine	CH_3_Hg^+^/Hg^2+^: 0.1 nM	1–1000 nM	-	water	[20]
AIE-based fluorescence	tetraphenylethylene–monoboronic acid	CH_3_Hg^+^/Hg^2+^: 0.12 ppm	0.6–30 ppm	-	Fish muscle	[31]
Colorimetric	DNA-Templated Ag–Au nanoparticles synthesis	0.5 μM	0–200 μM	tolerate with 50-fold Hg^2+^	Fish muscle	[21]
Colorimetric	DNA-AuNPs	5 nM	20–500 nM	tolerate with 1-fold Hg^2+^	Lake water	This work

^a^ cleanup using C18 cartridges.

**Table 3:** Detection of CH_3_Hg^+^ in real water samples (n = 3).

Water Sample	Added (nM)	Mean Found (nM)	Mean Recovery (%)
1	20	19.1 ± 0.9	95.5%
2	50	46.8 ± 2.3	93.6%
3	100	102.1 ± 3.7	102.1%

## Supplementary Materials

The following are available online at http://www.mdpi.com/1424-8220/18/8/2679/s1, Figure S1. Chromogenic reaction of TMB; Figure S2. UV–vis spectra of citrate-stabilized AuNPs + TMB-H_2_O_2_ reaction solution.
