# Erratum: Xie, Z.-J.; Bao, X.-Y.; Peng, C.-F. Highly Sensitive and Selective Colorimetric Detection of Methylmercury Based on DNA-Functionalized Gold Nanoparticles. *Sensors* 2018, *18*, 2679.

**DOI:** 10.3390/s18093135

**Published:** 2018-09-17

**Authors:** Zheng-Jun Xie, Xian-Yu Bao, Chi-Fang Peng

**Affiliations:** 1 School of Food Science and Technology, Jiangnan University, Wuxi 214122, China; xiezj@jiangnan.edu.cn (Z.-J.X.); 6150111037@vip.jiangnan.edu.cn (X.-Y.B.); 2 State Key Laboratory of Dairy Biotechnology, Shanghai Engineering Research Center of Dairy Biotechnology, Dairy Research Institute, Bright Dairy & Food Co., Ltd., Shanghai 200436, China; 3 Shenzhen Academy of Inspection and Quarantine, Shenzhen 518045, China

The authors wish to make the following corrections to their paper [1]: 

The order of the author Zheng-Jun Xie’s affiliations was incorrect in our published paper in Sensors [1]. Therefore, it is corrected from Z.-J.X. 

(^1^ State Key Laboratory of Dairy Biotechnology, Shanghai Engineering Research Center of Dairy Biotechnology, Dairy Research Institute, Bright Dairy & Food Co., Ltd., Shanghai 200436, China

^2^ School of Food Science and Technology, Jiangnan University, Wuxi 214122, China) to 

(^1^ School of Food Science and Technology, Jiangnan University, Wuxi 214122, China

^2^ State Key Laboratory of Dairy Biotechnology, Shanghai Engineering Research Center of Dairy Biotechnology, Dairy Research Institute, Bright Dairy & Food Co., Ltd., Shanghai 200436, China) 

The manuscript will be updated and the original will remain online on the article webpage.

The authors would like to apologize for any inconvenience caused.

## References

[1] Xie Z.-J., Bao X.-Y., Peng C.-F. (2018). Highly Sensitive and Selective Colorimetric Detection of Methylmercury Based on DNA Functionalized Gold Nanoparticles. Sensors.

