# Supplementary material for: Highly Sensitive and Selective Colorimetric Detection of Methylmercury Based on DNA Functionalized Gold Nanoparticles
Source: Sensors (Basel). 2018 Aug 15;18(8):2679. doi: 10.3390/s18082679 (PMC6111283; doi:10.3390/s18082679)
Supplement: Supplementary file 1 [file sensors-18-02679-s001.pdf]

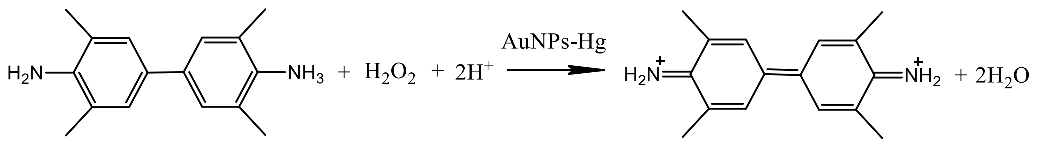

Figure S1. Chromogenic reaction of TMB

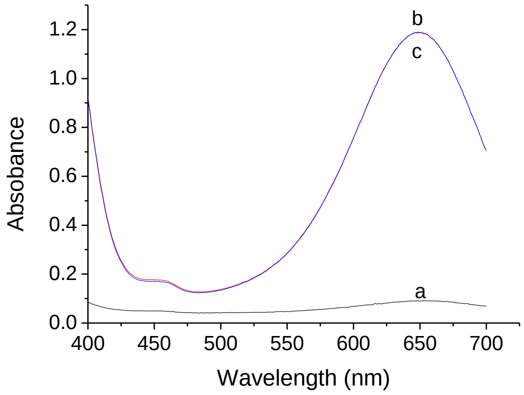

Figure S2. UV-vis spectra of citrate-stabilized AuNPs + TMB-H_2O_2 reaction solution (a) before and after incubated with (b) CH_3Hg^+ or (c) Hg^{2+} deposition.
